# Enzyme-labeled liquid-based cytology (ELLBC): a new noninvasive diagnostic method for bladder cancers

**DOI:** 10.1007/s00432-024-05613-9

**Published:** 2024-03-28

**Authors:** Chao Jiang, Xiang Li, Ruilong Chen, Yongliu Yang, Yi Wang

**Affiliations:** 1 Department of Urology, The Second Affiliated Hospital of Anhui Medical University, No. 678 Furong Road, Hefei, 230601 China; 2 Department of Pathology, Hefei Cancer Hospital, Chinese Academy of Sciences, No. 68 Yangqiao Road, Hefei, 230088 China; 3 Anhui Provincial Institute of Translational Medicine, Hefei, 230601 China

**Keywords:** Liquid-based cytology, Enzyme histochemical staining, Noninvasive diagnostic, Bladder cancer

## Abstract

**Background:**

Based on liquid-based cytology, we performed an enzyme histochemical staining using acid phosphatase as a marker and termed it ELLBC. The aim of this study was to investigate the value of ELLBC in the diagnosis of bladder cancer.

**Methods:**

Fifty patients who were initially diagnosed with suspected bladder cancers (hematuria or bladder irritation symptoms, urinary ultrasound suggestive of bladder mass) at the Second Affiliated Hospital of Anhui Medical University (Anhui, China) from January 2022 to December 2022 were selected as the study subjects, all of whom underwent ELLBC, CC, and histopathology Histopathology was used as the gold standard to calculate the diagnostic efficacy of ELLBC, CC and ELLBC combined with CC in bladder cancer.

**Results:**

Histopathological examination revealed 35 positive cases in 50 patients, including 15 cases of high-grade uroepithelial carcinoma (HGUC) and 20 cases of low-grade uroepithelial carcinoma (LGUC.) The sensitivity of ELLBC was 82.86%, the specificity was 93.33%, the positive predictive value (PPV) was 96.67%, the negative predictive value (NPV) was 70.00%, and the accuracy was 86.00%; CC had a sensitivity of 37.14%, specificity of 80.00%, PPV of 81.25%, NPV of 35.29%, and accuracy of 50%; ELLBC combined with CC had a sensitivity of 88.57%, specificity of 73.33%, PPV of 88.57%, NPV of 73.33%, and accuracy of 84.00%. The sensitivity and specificity of ELLBC were higher than that of CC, and the difference was statistically significant (*p* < 0.05), ELLBC combined with CC achieved higher sensitivity, but the diagnostic accuracy decreased. For clinical staging, the diagnostic accuracy was 86.36% for ELLBC and 40.91% for CC in patients in Stage I, and 90.91% for ELLBC and 36.36% for CC in patients in Stage II.

**Conclusion:**

ELLBC has high clinical application value for the diagnosis of bladder cancer and can provide new options and methods for the early screening of bladder cancer.

**Supplementary Information:**

The online version contains supplementary material available at 10.1007/s00432-024-05613-9.

## Introduction

Uroepithelial carcinoma (UC) is the most common type of bladder cancer, accounting for 90% of cases. It is a prevalent malignancy affecting the urinary tract. According to the American Cancer Society's Annual Review of Cancer Statistics 2022, bladder cancer ranks fourth in terms of the number of new cases among men and eighth in terms of the number of deaths among all cancers (Siegel et al. 2023).

Cystoscopy is considered the gold standard for diagnosing and monitoring bladder cancer. The diagnosis of bladder cancer relies on cystoscopy and histologic evaluation of tissue samples (Babjuk et al. 2022). However, cystoscopy is an invasive procedure that often leads to poor patient compliance. It can also cause discomfort, anxiety, and concerns about disease progression (Koo et al. 2017).

Biomarkers have been integrated into clinical decision-making for various cancers, including breast cancer (Chan et al. 2017). Consequently, there has been significant research interest in noninvasive urine tests with high diagnostic accuracy (Miyake et al. 2018; Costantini et al. 2021). However, despite the use of biomarkers, achieving the desired diagnostic rate remains a challenge. Therefore, cytology continues to be the gold standard for bladder cancer surveillance in many medical practices (Ng et al. 2021). Routine urine cytology has a sensitivity of 48% (16% for low-grade and 84% for high-grade) and a specificity of 86% (Yafi et al. 2015). However, these results may be influenced by the differentiated morphology and number of cells in the sample, and the test has lower sensitivity for cancers with low-grade superficiality, indicating certain limitations.

ELLBC utilizes enzyme histochemistry based on LBC to identify abnormally elevated acid phosphatase in bladder cancer cells. This method differs from traditional Papanicolaou staining and is currently employed for detecting exfoliated cells in pleural and abdominal fluid, as well as pericardial effusion (Li et al. 2018). ELLBC was developed to address the limitations of traditional smears, such as cloudy cells and blood contamination. In this study, we applied ELLBC for early noninvasive diagnosis of bladder cancer in a clinical setting and further validated its efficacy through a prospective cohort study. Additionally, we systematically evaluated the performance of this technique in diagnosing UC and compared it with routine urine exfoliative cytology.

## Methods

### Patients and samples

**Fig. 1:**
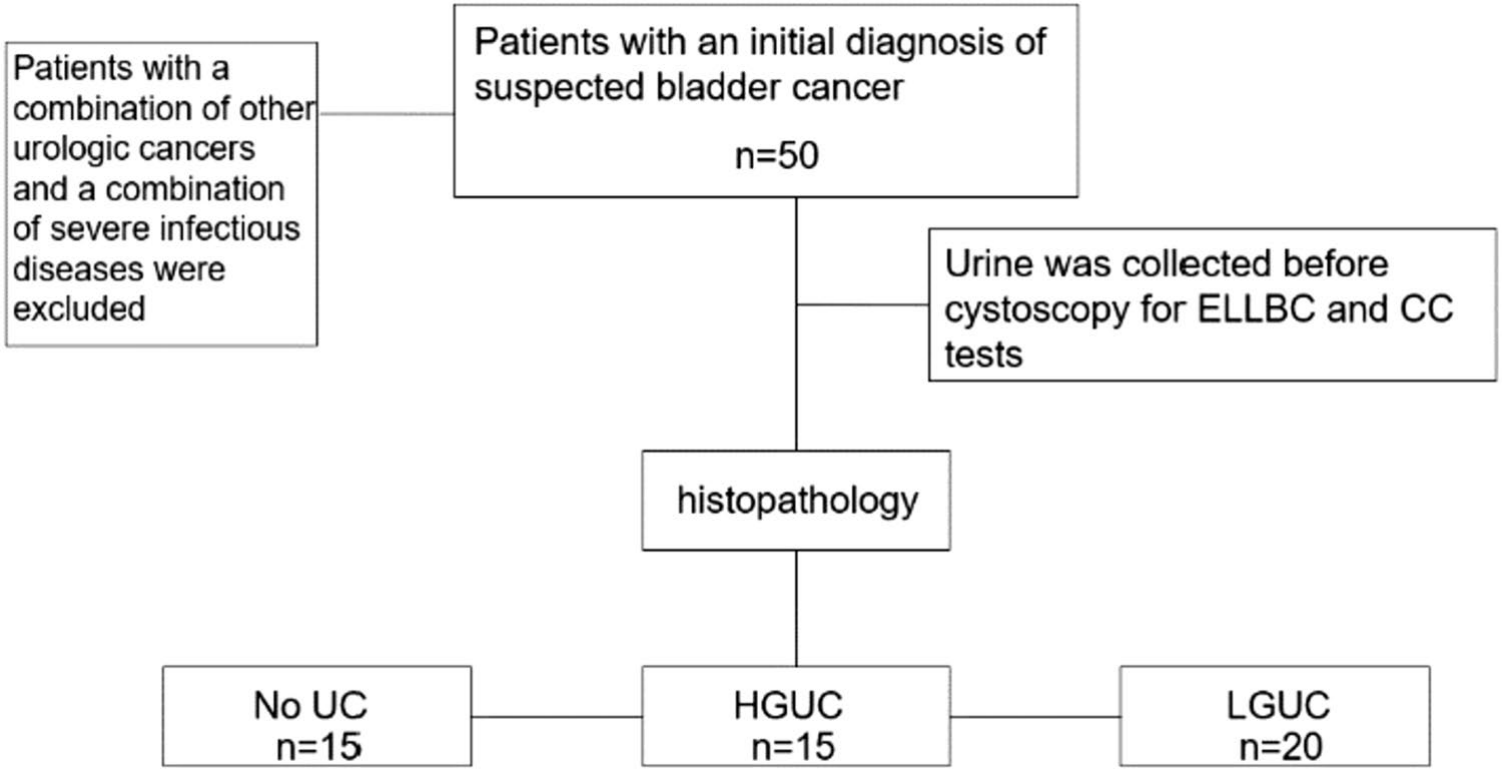
Inclusion criteria and histopathological results. *ELLBC* Enzyme-labeled liquid-based cytology, *CC* conventional urine cytology, *UC* Uroepithelial carcinoma, *HGUC* high-grade uroepithelial carcinoma, *LGUC* low-grade uroepithelial carcinoma)

Fifty patients who were initially diagnosed with suspected bladder cancers, based on symptoms such as hematuria or bladder irritation and urinary ultrasound suggestive of bladder mass, were selected from the Second Affiliated Hospital of Anhui Medical University (Anhui, China) between January 2022 and December 2022 (Patients with a combination of other urologic tumors and a combination of severe infectious diseases were excluded). Non-morning urine samples were collected from the patients, and ELLBC and CC tests were performed. Before participating in the study, all patients signed a consent form that allowed clinicians to access their medical records for clinical information. The study protocol was approved by the Ethics Committee of the Second Affiliated Hospital of Anhui Medical University (Ethics Approval No. YX2022-064). The process is illustrated in Fig. 1.

### Urine collection and preparation staining

**Fig. 2:**
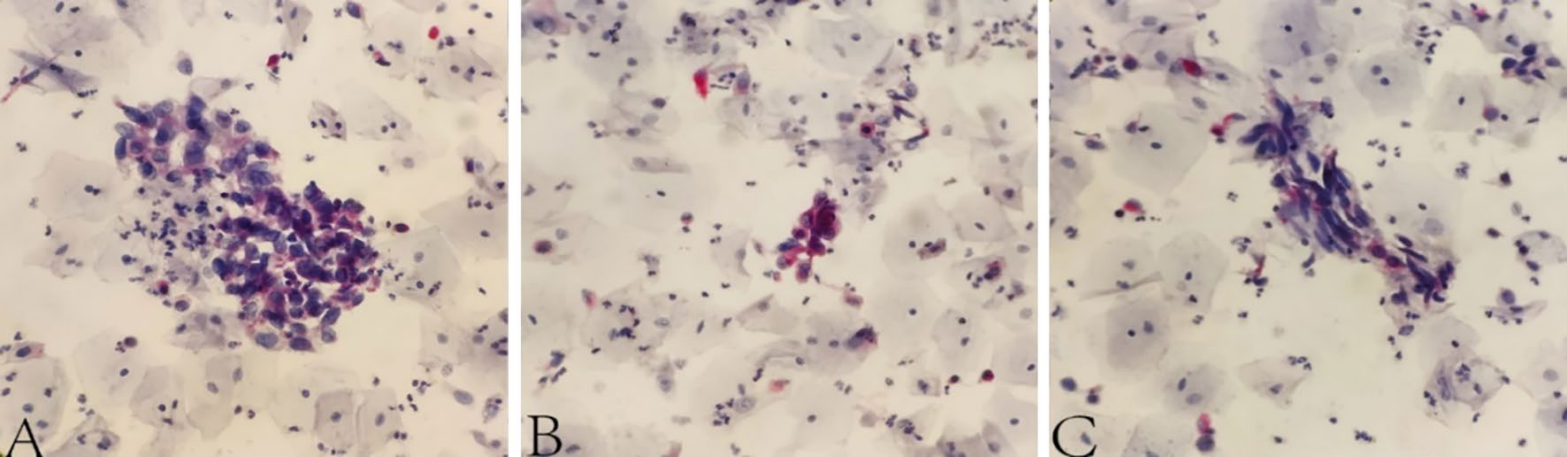
Representative images of LBC samples with enzyme histochemical staining **A**–**C** are three views of a smear showing anomalous cells and abnormal red staining). Degree of cytoplasmic red dye hint of acidic phosphatase expression. The magnification of the figures is × 200

After admission, 100 mL of fresh urine (non-morning urine) was collected from the patients using enzyme-free EP tubes for examination by ELLBC and CC, respectively. Only one urine sample is required for ELLBC and three samples are required on three consecutive days for CC. The CC slide preparation involved double centrifugation of the sediment obtained from the urine samples, followed by Papanicolaou staining. The slides were then sent to the pathologist for microscopic reading after completion of the smear and transparency operations. For ELLBC, 100 mL of collected fresh urine was centrifuged at 2000 rpm for 10 min, washed with enzyme-labeled liquid-based cytological preservation solution, and prepared until the cells were partially dry. The partially dry cell smear was then fixed in a fixing solution at room temperature for 4 min to remove red blood cells, rinsed with tap water for 2 min, placed in a staining solution of acid phosphatase at 37 °C for 5–10 min, rinsed with running water for 2 min, dyed with nuclear redyeing solution for 5 min, rinsed again with running water for 2 min, and finally sealed with neutral gum after thorough air drying. The cytology reports were completed using the Paris system and the results were categorized as positive or negative. Positive results indicated confidence or suspicion of malignancy, while negative results indicated findings other than malignancy, such as inflammation or benign conditions. The final diagnosis of the patient was based on the histopathological findings. The dyeing process of ELLBC is shown in Figure S1, and the composition of the acid phosphatase stain solution is described in Table S1. The cytology reports were completed using the Paris system, with results categorized as positive or negative. Positive results indicated confidence or suspicion of malignancy, while negative results indicated findings other than malignancy, such as inflammatory or benign conditions. In all cases, the diagnosis was confirmed by the same pathologist, and the final diagnosis of the patient was based on the histopathological findings, which served as the gold standard. Figure 2 shows a representative image of the staining.

### Data analysis

Pathologic findings, ELLBC findings and CC findings were collected for 50 patients. The diagnosis of ELLBC relies on morphologic observation and specific red staining of the cytoplasm, and the diagnosis of CC can only be determined by observation of the nucleoplasmic ratio of the cells. The results were considered positive if abnormal cells were observed in one sample in the ELLBC group and one of three samples in the CC group. The sensitivity, specificity, positive predictive value (PPV), negative predictive value (NPV), and accuracy of ELLBC and CC tests in diagnosing bladder cancer were calculated against the pathologic findings, which were considered the gold standard. The calculation formula can be found in Supplementary Table S2. Additionally, the sensitivities of the two cytologic techniques were determined for different grades of uroepithelial carcinoma in the bladder. The chi-square test was used to analyze the differences in underestimation and overestimation rates between CC and ELLBC in detecting lesions. Statistical analysis was performed using SPSS (version 26, SPSS Inc., Chicago, IL, USA). A significance level of *p* < 0.05 was used to determine statistical significance.

## Results

**Fig. 3:**
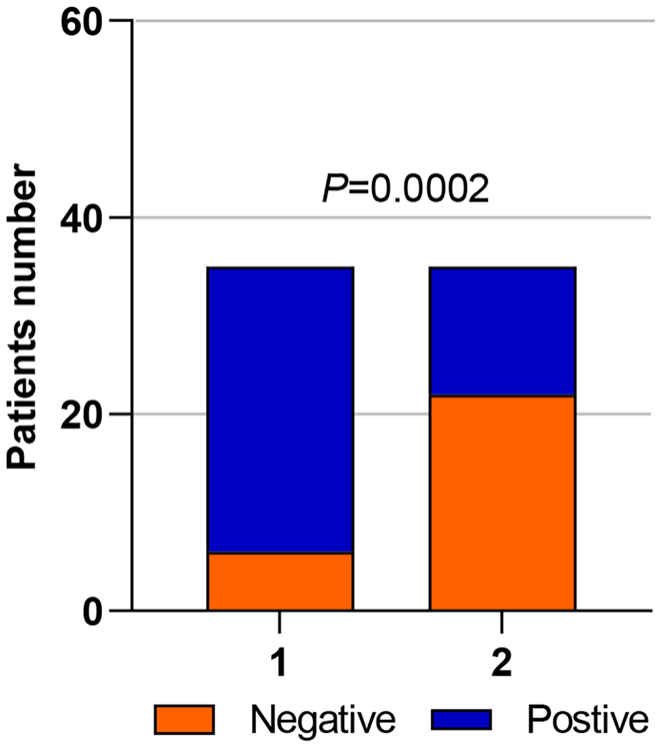
ELLBC and CC test results.The final pathological diagnosis was used to determine (group 1:ELLBC; group 2:CC). Chi-square test shows a significant difference between the two (*p* = 0.0002)

**Fig. 4:**
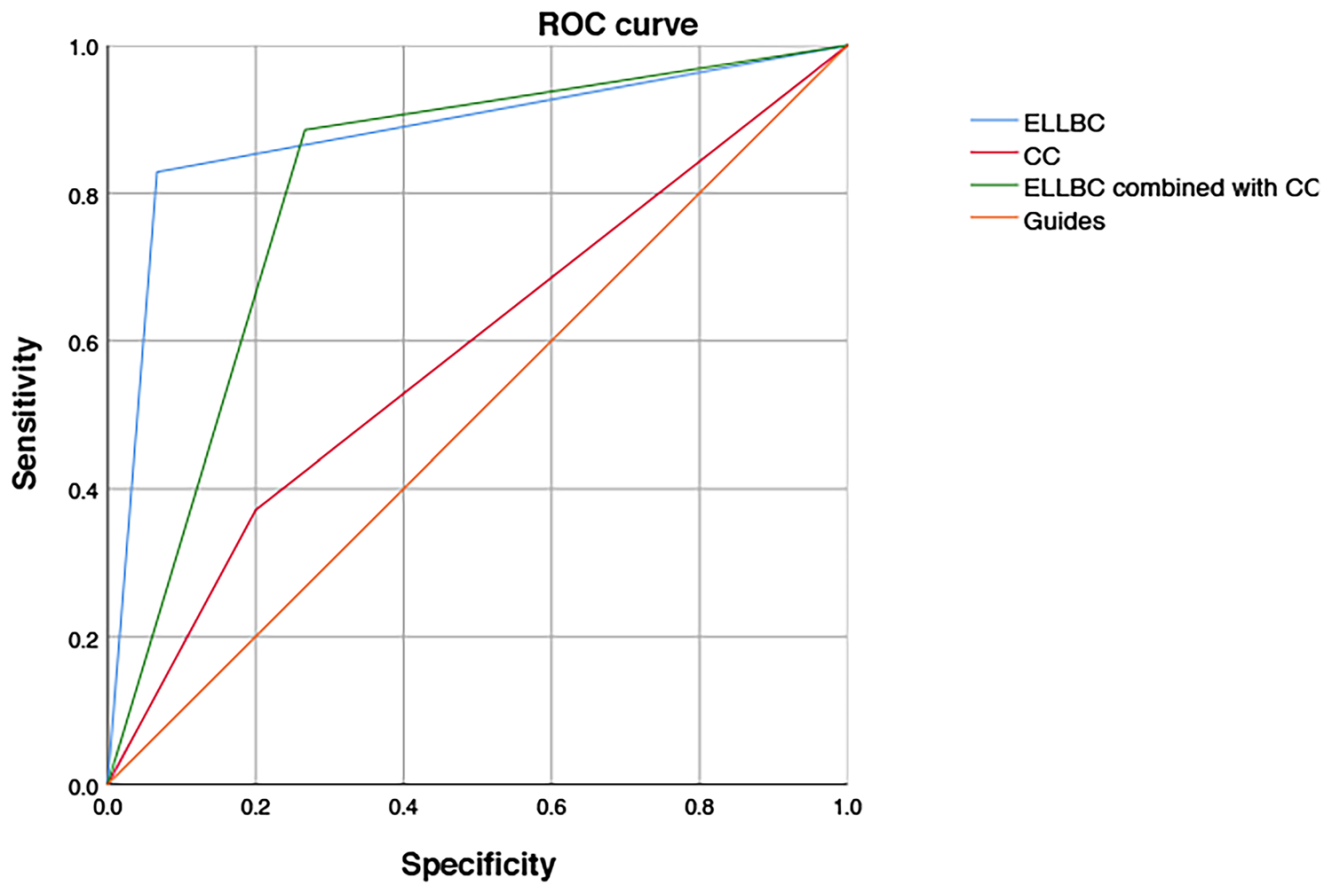
The ROC curve results are drawn

Pathological diagnosis showed that there was a significant difference in the detection rate of bladder tumors between ELLBC and CC groups (Fig. 3). Pathological diagnosis showed that there was a significant difference in the detection rate of bladder tumors between ELLBC and CC groups; There was no significant difference in the detection of high-grade tumors between the two methods (Table 1A). The sensitivity of ELLBC was 82.86%, the specificity was 93.33%, the PPV was 96.67%, the NPV was 70.00%, and the accuracy was 86.00%; CC had a sensitivity of 37.14%, specificity of 80.00%, PPV of 81.25%, NPV of 35.29%, and accuracy of 50%; ELLBC combined with CC had a sensitivity of 88.57%, specificity of 73.33%, PPV of 88.57%, NPV of 73.33%, and accuracy of 84.00% (Table 1B). For clinical staging, ELLBC and CC showed a significant difference in diagnosis between stages I and II, but not in stages III and IV (Table 1C). We collected the diagnostic results of ELLBC and CC and plotted the Receiver Operating Characteristic (ROC) Curve of ELLBC, the resulting area under the curve was 0.881 for ELLBC (95% CI = 0.776–0.986), 0.586 for CC (95% CI = 0.417–0.754), and 0.810 for ELLBC combined with CC (95% CI = 0.663–0.956) (Fig. 4).

**Table 1:** Test results (A) and diagnostic performance (B-C) of ELLBC and CC

A				
Parameter	CC	ELLBC	Frequency	*P*
Urine malignant cell detection/case (%)				
	13 (37.14)	29 (82.86)	*n* = 35	0.0007
Low-grade cancers detection/case (%)				
	4 (20)	15 (75)	*n* = 20	0.0012
High-grade cancers detection/case (%)				
	9 (60)	11 (73.33)	*n* = 15	0.6999
Patients with persistent hematuria detection/case (%)				
	4 (13.33)	16 (53.33)	*n* = 30	0.0022

B			
	CC (95%CI)	ELLBC (95%CI)	ELLBC + CC (95%CI)
Accuracy (%)	50.00	86.00	84.00
Sensitivity (%)	37.14 (23.17–53.66)	82.86 (67.32–91.90)	88.57 (74.05–95.46)
Specificity (%)	80.00 (54.81–92.95)	93.33 (70.18–99.66)	73.33 (48.05–89.10)
PPV (%)	81.25 (56.99–93.41)	96.67 (83.33–99.83)	88.57 (74.05–95.46)
NPV (%)	35.29 (21.49–52.09)	70.00 (48.10–85.45)	73.33 (48.05–89.10)

C				
Clinical Stage	CC	ELLBC	Frequency	*P*
Stage I (%)	9 (40.91)	19 (86.36)	22	0.004
Stage II (%)	4 (36.36)	10 (90.91)	11	0.0237
Stage III (%)	1 (100.00)	1 (100.00)	1	–
Stage IV (%)	1 (100.00)	1 (100.00)	1	–

### Smears prepared by ELLBC and CC

**Fig. 5:**
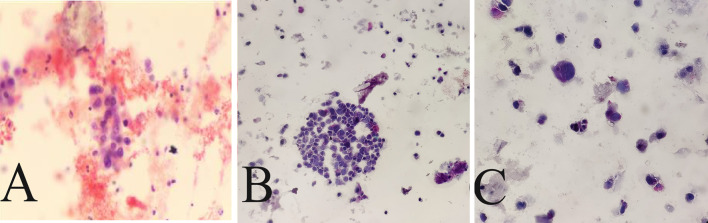
**A** shows that the background of the smear obtained from the CC examination of this patient is more turbid; **B** and **C** show that the background of the smear obtained from the ELLBC examination of this patient is clearer

The study's findings revealed that in the urine cytology of patients with chronic haematuria, the CC preparation's background was more muddy (Fig. 5).

## Discussion

Bladder cancer screening and post-treatment surveillance still rely on conventional urine cytology combined with cystoscopy, but cystoscopy shows low sensitivity in the face of low-grade uroepithelial cancers and small lesions (Miyake et al. 2021), whereas the diagnostic sensitivity and specificity of conventional urine cytology are low and show a large discrepancy between the detection of low-grade and high-grade uroepithelial cancers (Babjuk et al. 2022). The obvious disadvantage of bladder cancer surveillance is the expensive financial burden, which accounts for 5.78% of all out-of-pocket costs for cancer patients in the United States (Schafer et al. 2023),in the European Union this value is 3% (Leal et al. 2016). Therefore, an increase in the diagnostic rate of cytology is of significant significance for the reduction of healthcare costs for bladder cancer patients.

The most criticized drawback of conventional urine cytology is its low detection rate (20–50%) (Yafi et al. 2015), and similar results were obtained in our study, which were more pronounced in low-grade uroepithelial carcinomas, which may be attributed to the fact that low-grade cancers have a similar cellular morphology to the normal uroepithelium (Barkan et al. 2016).

Another possible cause of the CC detection rate is cellular or blood contamination; urine collected from patients with persistent hematuria has lower sensitivity for cytology and the presence of a large number of atypical cells that need to be interpreted (Tan et al. 2019; Konety et al. 2019), which adversely affects the interpretation of malignant cells,urine samples accompanied by a large amount of inflammatory infiltrate, cellular degeneration, or heavy bleeding may lead to overdiagnosis or an underdiagnosed basis for the diagnosis (Sweeney et al. 2005). In contrast, liquid-based cytology preparation can exclude blood cell interference (Remmerbach et al. 2017; Xu et al. 2018) and obtain a clearer background (Chou et al. 2015), and our use of acid phosphatase as an enzyme marker also yielded a more favorable result for the interpretation of malignant cells, as shown in Fig. 3, which shows that ELLBC has a clear advantage over CC in the diagnosis of patients with persistent hematuria.

Six urinary biomarkers approved by the U.S. Food and Drug Administration for the diagnosis or surveillance of bladder cancer have been evaluated in recent studies: quantitative or qualitative nuclear matrix protein 22 (NMP22), qualitative or quantitative bladder cancer antigen (BTA), fluorescence in situ hybridization (FISH), and fluorescence immunohistochemistry (ImmunoCyt), with a sensitivity range of 0.57–0.82, and specificity ranging from 0.74 to 0.88 (Tabayoyong and Kamat 2018; Goutas et al. 2021). The sensitivity of ELLBC reached the upper limit of conventional assays, and the specificity was superior to conventional assays.

Enzyme histochemical staining is a method to show the activity and location of endogenous enzymes in tissues or cells on slices or smears by using the characteristics of intracellular enzymes to catalyse substrates. Its characteristics for in situ detection enzyme expression and enzyme activity rather than itself. As early as 1952, the histochemistry of alkaline phosphatase was confirmed by azo-dye coupling method (Grogg and Pearse 1952). Related studies in the past few years have shown that enzyme histochemistry staining can assist in monitoring the therapeutic effect of radiofrequency ablation for primary breast cancer (Seki et al. 2011). The color reaction of azo dye can obtain clearer antigen cell localization than immunofluorescence technology. Therefore, immunostaining with enzyme-labeled probes can be used in complex situations that require dual antigen localization (Tsutsumi 2021). In urine cytology, it may be simpler and of higher economic value to use azo dye-coupled method to monitor abnormally elevated acid phosphatase in bladder cancer cells.

Although ELLBC had better sensitivity when combined with CC, its area under the curve was still smaller than that of ELLBC, and ELLBC yielded better specificity when compared with CC and sensitivity, and better sensitivity was obtained when the two were combined for monitoring, but the overall efficiency was still not as good as with ELLBC alone.

Our study ultimately collected urine samples from 50 patients, however, the cytological staining of the enzymatic labeling method has some limitations of its own, i.e., better results can be obtained by reading the film after the staining is completed, i.e., after the staining of the preparation is completed and stored at room temperature, the smears may show varying degrees of discoloration after 1–3 months, which may appear to have some impact on the determination of the results. The final number of cases we included was only 50, which led us to realize that the clinical stages were mostly Stage I and Stage II, and a small number of patients were postoperative recurrences. This demonstrates the excellence of ELLBC performance in early screening. We will expand the caseload and focus on patients with different clinical stages and postoperative recurrence in subsequent studies to improve the applicability of ELLBC in clinical diagnosis.

One notable constraint of our study is its non-randomized trial design, which may have resulted in a lack of standardization regarding the number of passes for each method and a potential bias towards the smear method. Consequently, careful interpretation of the findings is warranted. Additionally, the study is limited by its small sample size and the absence of an on-site cytopathologist to evaluate sample adequacy during collection. Technical difficulties during sample preparation may have contributed to cellular issues, suggesting that ELLBC could offer improved applicability.

In conclusion, the ELLBC technique has a high sensitivity to bladder malignant cancers, and the application of this technique contributes to the early screening of bladder cancer, which may compensate for the low detection rate of conventional cytology. Cytology is a simple and inexpensive technique that can be used as an effective diagnostic tool for early screening of bladder cancer.

## Conclusion

The findings of this study demonstrate the potential value of enzyme-labeled liquid-based cytology technology as a noninvasive diagnostic tool for bladder cancer. However, it is important to note that there are still significant opportunities for further advancements in the cytologic diagnosis of bladder cancer. It is anticipated that this research will contribute to the enhancement of clinical decision-making in this field.

## Supplementary Information

Below is the link to the electronic supplementary material.

Supplementary file1 (DOCX 181 kb)

## Data Availability

In a suitable manner, we got pertinent data and materials from the corresponding author. Data regarding the study can be obtained by email from the corresponding author by wangyi2035@ahmu.edu.cn.

## Abbreviations

LBC: Liquid-based cytology
CC: Conventional cytology
UC: Urothelial cancer
HGUC: High-grade urothelial cancer
LGUC: Low-grade urothelial cancer
PPV: Positive predict value
NPV: Negative predict value
ACP: Acid phosphatase
ROC: Receiver operating characteristic
NMP22: Nuclear matrix protein 22
BTA: Bladder cancer antigen
FISH: Fluorescence in situ hybridization
ImmunoCyt: Fluorescence immunohistochemistry
