# Supplementary material for: Enzyme-labeled liquid-based cytology (ELLBC): a new noninvasive diagnostic method for bladder cancers
Source: J Cancer Res Clin Oncol. 2024 Mar 28;150(3):169. doi: 10.1007/s00432-024-05613-9 (PMC10978622; doi:10.1007/s00432-024-05613-9)
Supplement: Supplementary file 1 — Supplementary file1 (DOCX 181 kb) [file 432_2024_5613_MOESM1_ESM.docx]

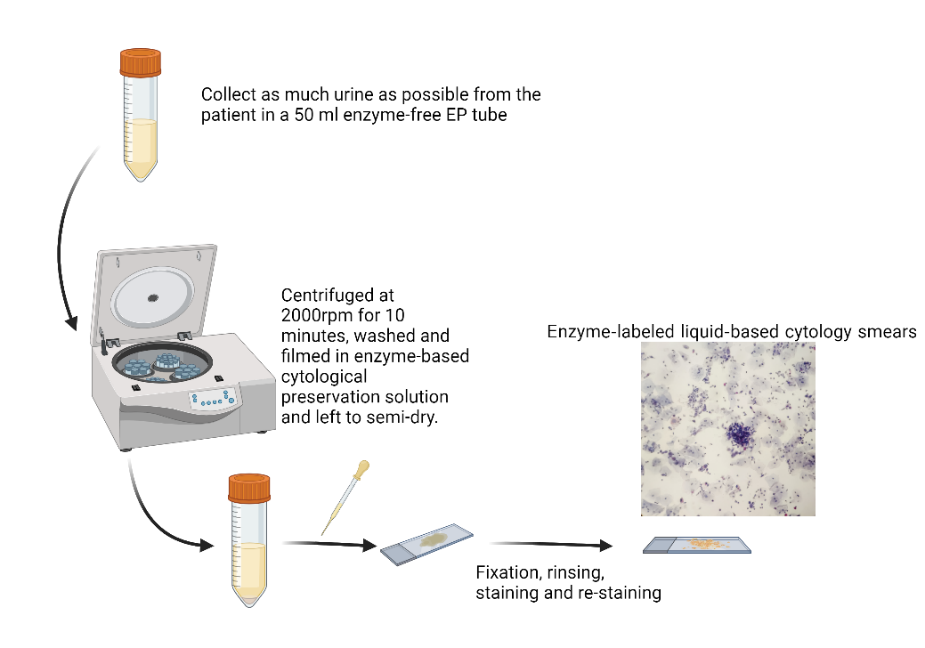
Supplementary Fig S1

Figure S1: The specific steps and staining examples of enzyme histochemical staining

Supplementary Table S1

Component of acid phosphatase stain solution

| acid phosphatase stain solution | 25ml distilled water |
| --- | --- |
|  | 0.5ml magenta hydrochloride solution |
|  | 0.5ml sodium nitrite solution |
|  | 1.5ml naphthol solution |
|  | 1.5ml sodium acetate solution |

Figure 2: After staining in acid phosphatase dye solution, red granular precipitates are formed in cells with acid phosphatase activity

Supplementary Table S2

|  |  | Histopathology | |  |
| --- | --- | --- | --- | --- |
|  |  | Malignant | Benign | Total |
|  |  |  |  |  |
| ELLBC/CC/ELLBC+CC | Suspicious of malignancy/ Malignant | a | b | m+ |
|  | Benign | c | d | m- |
|  | Total | n+ | n- |  |

Formulas for calculating cytology performance.

Accuracy : a+d/a+b+c+d

Sensitivity : a/n+

Specificity : d/n-

Positive Predictive Value (PPV) : a/m+

Negative Predictive Value (NPV) : d/m-
